# A dataset on the use of online video by students at the in-video level

**DOI:** 10.1016/j.dib.2025.112132

**Published:** 2025-10-13

**Authors:** César Córcoles, Germán Cobo Rodríguez, Ana-Elena Guerrero-Roldán, M. Antonia Huertas

**Affiliations:** a Universitat Oberta de Catalunya, Rambla del Poblenou, 156, 08018, Barcelona, Spain; b Universitat Autònoma de Barcelona, Applied Pedagogy Department, Edifici G6, Campus de la UAB, 08193 Bellaterra (Cerdanyola del Vallès), Spain

**Keywords:** Learning analytics, Video-based learning, Clickstream analytics, Educational data mining

## Abstract

The present manuscript describes a dataset containing learning analytics data for the playback of learning materials in video format in different college courses in the STEM field, across a period of ten years. It can be used to test hypothesis and tools regarding the use of video in different learning environments, and should be of interest to the learning analytics and educational data mining communities. It can also be of help to teachers and other stakeholders in the educational process to take decisions based on learners actions when playing videos. It consists of data for 35 different videos, with a total of 40,453 sessions, and 313,724 records. The videos are accompanied by their timestamped transcription, both in the original language and their translation into English.

Specifications Table

**Table:** 

Subject	Social science
Specific subject area	Learning analytics (the measurement, collection, analysis, and reporting of data about learners and their contexts, for the purposes of understanding and optimizing learning and the environments in which it occurs).
Type of data	Raw, files to process raw data.
Data collection	Data were collected by embedding JavaScript code in different web pages containing video-based learning resources. Said code intercepted actions taken by students while watching those video-based learning resources and sent it to a server to be stored. For this purpose, JavaScript on the client side and PHP on the server side were used. Some data processing was carried out in Python. The transcription and translation of the videos was outsourced to a company offering those services.
Data source location	https://www.uoc.edu
Data accessibility	Repository name: Zenodo Data identification number: 10.5281/zenodo.14221667 Direct URL to data: https://doi.org/10.5281/zenodo.14221667
Related research article	Córcoles, C., Cobo, G., & Guerrero-Roldán, A.-E. (2021). The Usefulness of Video Learning Analytics in Small Scale E-Learning Scenarios. Applied Sciences, 11(21), 10,366. https://doi.org/10.3390/app112110366.

## 1 Value of the Data

• The dataset we offer in this publication can be used by experts in the domains of learning analytics (LA) and educational data mining (EDM), on one hand, as a tool to test hypothesis and tools regarding video-based learning without the need for the process of data collection and, on the other hand, as an orientation as to how best collect those data if necessary.
• In our research, the dataset has proved useful, when visualized as in Córcoles et al. [1], and especially in Córcoles et al. [2], to (i) confirm or deny instructors hypothesis regarding difficulty and cognitive load for different learning resources, or (ii) to detect navigational patterns that occur for certain student profiles or at certain moments in the course, which present an opportunity to offer navigational help to improve student experience, to offer personalised content to students presenting one of these navigational patterns, or to better structure the presentation of information in a future version of a video.
• While the fields of LA and EDM have, of course, studied how students interact with learning resources (see Vieira et al. for examples [12]), they are often more concerned with how students deal with sequences of learning materials than with how they interact with a single learning resource as a venue for the teaching and learning process. But online video, and the digital trace it leaves, allows us to dig down and operate at a lower level of granularity.
• Currently published research focusing on clickstream analytics (Kim et al. [3], Giannakos et al. [4], Kleftodimos et al. [5], Lau et al. [6], Shoufan et al. [7], Min et al. [8], Hasan et al. [9], Hu et al. [10], Mubaraket al. [11]) has been more concerned with providing information for the improvement of educational videos than with analysing how individual students learn from them and trying to detect behavioural patterns and intervene on their learning, which is an expected result from the publication of the dataset).
• To the best of our knowledge, no other comparable datasets are currently publicly available.

## 2 Background

The present dataset was created as one of the artefacts in a study to analyse how students learn from learning resources in video format. In order to carry out that study, it was necessary to collect anonymous data on all interactions that students have with those educational videos.

In the last few years there has been a movement to more use of online resources for teaching and learning, with the advent of online education, MOOCs, flipped classroom environments and other forms of education. There are numerous advantages to that move, but also some limitations. Firstly, most, if not all, of the feedback experienced teachers obtain in face-to-face teaching situations and use to diagnose how students are learning from visual cues is lost when students use online resources. It is possible to capture some of that lost information because consuming online video leaves a trace: we can record when a student has started watching a video, or the moments when he/she paused it or skipped over a part of it, for example. It seems reasonable to expect that kind of information could make it possible to give teachers some feedback regarding the student's learning process (Córcoles et al. [1]).

The data was not made publicly available at the time of the study because internal data from the learning management system was still available, and it could have been used to deanonymize the released data. That is no longer the case.

## 3 Data Description

We have created a single table MySQL/MariaDB database. Its fields are as follows:

• Identifier
  Column name: id
  Type: bigint(20) NOT NULL AUTO_INCREMENT
  Description: It contains an (autoincrementing) identifier for each record in the table. It is an unsigned long integer.
• Initial timestamp
  Column name: timestamp_inicial
  Type: varchar(255) NOT NULL
  Description: It contains the timestamp of the moment in which the browser loads the web page. It is generated in the browser (using the JavaScript command sequence var t_ini = new Date(); t_ini.setTime(Date.now()); when the page is loaded) and then converted to the RFC 7231 format using the toUTCString() method of the JavaScript Date object. This is used (in conjunction with a random key) to identify individual sessions in an anonymous way.
• Random key
  Column name: random
  Type: varchar(255) NOT NULL
  Description: When the browser accesses the web page containing the video, it generates a random floating point number between 0 and 1 with approximately uniform distribution over that range (using the JavaScript command var rnd_key = Math.random();). This number is only of interest if two different sessions were to start at the precise same initial timestamp.
• Video
  Column name: video
  Type: varchar(255) NOT NULL
  Description: It is a string containing an arbitrarily created identifier for the video the user is playing. The list of videos is contained in Tables 1, Table 2, Table 3, below.

**Table 1:** Non-relational database videos content descriptions and URLs. Transcriptions and translations for videos marked with (*) have been created by a company offering said services. The rest of transcriptions have been created by using Whisper [19].

Video identifier	Description & URL
nosqlintro	An introduction to NoSQL databases http://cimanet.uoc.edu/videos/bbdd/intro.html
nosqlpersistencia (*)	Polyglot persistance http://cimanet.uoc.edu/videos/bbdd/persistencia.html
nosqlmagregamot (*)	Aggregation models. Motivation http://cimanet.uoc.edu/videos/bbdd/magregamotivacion.html
nosqlmagregacar (*)	Aggregation models. Features http://cimanet.uoc.edu/videos/bbdd/magregacaracteristicas.html
magregatip	Aggregation models. Types http://cimanet.uoc.edu/videos/bbdd/magregatipos.html
mgrafo	Graph model http://cimanet.uoc.edu/videos/bbdd/modelografo.html
distrintro (*)	Introduction to distributed databases http://cimanet.uoc.edu/videos/bbdd/distrintro.html
distrarq	Distributed databases. Architectures http://cimanet.uoc.edu/videos/bbdd/distrarq.html
distrdiseno	Distributed databases. Design http://cimanet.uoc.edu/videos/bbdd/distrdiseno.html
distracid	Distributed databases. ACID model http://cimanet.uoc.edu/videos/bbdd/distracid.html
distrCAPBASE	Distributed databases. CAP theorem and BASE model http://cimanet.uoc.edu/videos/bbdd/distrCAPBASE.html
mapreduce	Distributed databases. Map reduce framework http://cimanet.uoc.edu/videos/bbdd/mapreduce.html
riak	Key-value database example: Riak http://cimanet.uoc.edu/videos/bbdd/riak.html
mongodb	Document database example: Mongo http://cimanet.uoc.edu/videos/bbdd/mongodb.html
neo4j	Graph database example: Neo4j http://cimanet.uoc.edu/videos/bbdd/neo4j.html
usoneo4j	Neo4j VM use http://cimanet.uoc.edu/videos/bbdd/usoneo4j.html
mongoCREA (*)	MongoDB guided practical exercise (I) http://cimanet.uoc.edu/videos/bbdd/mongoCREA.html
mongoCONS	MongoDB guided practical exercise (II) http://cimanet.uoc.edu/videos/bbdd/mongoCONS.html
mongoACT	MongoDB guided practical exercise (III) http://cimanet.uoc.edu/videos/bbdd/mongoACT.html
instNeo4J (*)	Neo4j installation http://cimanet.uoc.edu/videos/bbdd/instNeo4J.html
restNeo4J	Neo4j Twitter database restoration http://cimanet.uoc.edu/videos/bbdd/restNeo4J.html
intronsqles	An alternative introduction to NoSQL databases https://vimeo.com/149250649

**Table 2:** Relational database videos content descriptions and URLs. Transcriptions and translations for videos marked with (*) have been created by a company offering said services. The rest of transcriptions have been created by using Whisper [19].

Video identifier	Description & URL
05585_01 (*)	Database design. Conceptual design. Catalan language http://cimanet.uoc.edu/videos/5585/01ca.html
75,585_01 (*)	Database design. Conceptual design. Spanish language http://cimanet.uoc.edu/videos/5585/01es.html
05585_02 (*)	Database design. Logic design. Catalan language http://cimanet.uoc.edu/videos/5585/02ca.html
75,585_02 (*)	Database design. Logic design. Spanish language http://cimanet.uoc.edu/videos/5585/02es.html
02865_01	Database design. Conceptual design. Catalan language http://cimanet.uoc.edu/videos/5585/02ca.html
02965_01	Database design. Conceptual design. Spanish language http://cimanet.uoc.edu/videos/5585/01_2965.html
02865_01	Database design. Logic design. Catalan language http://cimanet.uoc.edu/videos/5585/01_2865.html
02965_02	Database design. Logic design. Spanish language http://cimanet.uoc.edu/videos/5585/02_2965.html

**Table 3:** Statistics videos content description. All videos available at http://cimanet.uoc.edu/UOCEstadistica2. Transcriptions and translations for videos marked with (*) have been created by a company offering said services. The rest of transcriptions have been created by using Whisper [19].

Video identifier	Description
estadistica1 (*)	Normal distribution fundamentals. Catalan language
estadistica2	Normal distribution advanced operations. Catalan language
estadistica3	Student’s T distribution. Catalan language
estadistica4 (*)	Hypothesis tests. Catalan language
estadistica1p	Normal distribution fundamentals (video includes a navigation menu linking to different sections). Catalan language
• Action
  Column name: accion
  Type: varchar(255) NOT NULL
  Description: It contains one of six possible verbs: pageloaded (the web page containing the video is loaded, will appear at most once per session), play (the play button was pressed), seek (the user clicked or dragged on the video's timeline to move to a different point in the video), pause (the pause button was pressed), clickindex (an item on an index for a video was pressed; this results on the playhead moving to the position indicated by the index item), navigatedaway (the user browsed to a different webpage or closed the browser's window). The pageloaded action can be considered redundant, as it contains the same information that the initial timestamp field contains for any videos in which at least another action is recorded, and it has not been recorded for some of the videos, as detailed in Table 1, Table 2, Table 3 below. Some of the verbs take an additional parameter, stored in the params field, documented below.
• Action timestamp
  Column name: timestamp_accion
  Type: varchar(255) NOT NULL
  Description: It contains a timestamp for the moment in which the action was performed. It is generated in the browser (using the JavaScript command sequence var t_ara = new Date(); t_ara.setTime(Date.now()); when the action is recorded) and then converted to the RFC 7231 format using the toUTCString() method of the JavaScript Date object.
• Parameters
  Column name: params
  Type: varchar(255) NOT NULL
  Description: it contains any necessary parameters for the performed action. The pageloaded action takes no parameters. The play, seeked and paused actions take the moment in the video the playhead is at when the action takes place as a parameter. This is generated using the this.CurrentTime() method (as generated by the Popcorn.js JavaScript library in the browser). The clickindex action takes the time in the video the action jumps to as a parameter.

The MySQL command sequence used to create the database is as follows:

**Table:** 

DROP TABLE IF EXISTS `interacciones`;
CREATE TABLE `interacciones` (
`id` bigint(20) NOT NULL AUTO_INCREMENT,
`timestamp_inicial` varchar(255) NOT NULL,
`random` varchar(255) NOT NULL,
`video` varchar(255) NOT NULL,
`timestamp_accion` varchar(255) NOT NULL,
`accion` varchar(255) NOT NULL,
`params` varchar(255) NOT NULL,
PRIMARY KEY (`id`)
) ENGINE=MyISAM AUTO_INCREMENT=319,165 DEFAULT CHARSET=latin1;

The CSV file, being a straight conversion of the SQL table, has the same format and has been generated by using (https://github.com/yashsmehta/mysqldump-to-csv). A second CSV file, dump_headers.csv, contains the same information and adds a first line containing the column names for convenience. The first R file, dump.RData, consists of a single R object, a data frame, called dump. It has been generated by importing the CSV file, assigning it names to match the original SQL table structure and then creating a new "replayID" column, assigning a new id to every new combination of the columns page_loaded and rnd_key. The file can be opened using the following command in R:

**Table:** 

load(“dump.Rdata”)

Finally, a dump.parquet file in Adobe Parquet format has been created from the CSV file using uvx –with pandas –with pyarrow ipython and then the command sequence

**Table:** 

In [1]: import pandas as pd
In [2]: archivo = pd.read_csv('dump.csv')
In [3]: archivo.to_parquet('dump.parquet')

A second file in Adobe Parquet format has been included, containing column names in the first column. In order to access the Adobe Parquet files using R, the “arrow” software must be installed. After installation, the following command can be used to import the data to a dataframe:

**Table:** 

dump_frame <- read_parquet(dump.parquet)

A commented fragment of the CSV file is included in the Experimental design, materials and methods section.

• The dataset is composed of data from 30 different videos, some of which are presented in different versions. All videos are in Spanish or Catalan, with original language transcriptions and English translations provided in SRT format. The list of videos is contained in Tables 1, Table 2, Table 3, below.
• The main part of the dataset is composed of data from 22 videos on non-relational databases (intronosqles, nosqlintro, nosqlpersistencia, nosqlmagregamot, nosqlmagregacar, magregatip, mgrafo, distrintro, distrarq, distrdiseno, distracid, distrCAPBASE, mapreduce, riak, mongodb, neo4j, usoneo4j, mongoCREA, mongoCONS, mongoACT, instNeo4J, restNeo4J). These videos are in Spanish.
• Additionally, data from 4 different videos on statistics have been included in the dataset (estadistica1, estadistica2, estadistica3, estadistica4) in the Catalan language, with a fifth video (estadistica1p) being a variant of the first one (estadistica1), where it has been presented to students with a navigation menu linking to the different sections in the video.
• Finally, the dataset includes data from 8 versions of 4 videos on relational databases (05585_01, 75,585_01, 05585_02, 75,585_02, 02865_01, 02965_01, 02865_02, 02965_02). Videos are in Spanish ('75,585′ and '02,865′ filenames) and in Catalan ('05,585′ and '02,965′ filenames).
• A numerical summary of the number of sessions, records and video length for each video is contained in Table 4, below.

**Table 4:** Numerical summary for videos in the dataset.

Video identifier	Sessions	Records	Video Length (mm:ss)
nosqlintro nosqlpersistencia (*) nosqlmagregamot (*) nosqlmagregacar (*) magregatip mgrafo distrintro (*) distrarq distrdiseno distracid distrCAPBASE mapreduce riak mongodb neo4j usoneo4j mongoCREA (*) mongoCONS mongoACT instNeo4J (*) restNeo4J	2882 2023 2768 2228 2175 1828 2310 2142 2022 1894 1835 1781 1609 1420 1536 157 1590 1107 708 988 1495	21,166 13,637 19,706 12,865 14,705 12,656 13,532 15,769 17,418 15,141 13,730 9677 9766 8296 10,752 2447 12,831 5306 1902 6066 8001	25:00 18:41 17:32 11:09 23:32 38:24 12:41 21:21 25:26 49:25 36:07 30:46 48:34 30:18 60:34 8:10 17:33 23:16 15:03 7:47 6:36
estadistica1 (*) estadistica1p estadistica2 estadistica3 estadistica4 (*) 05585_01 (*) 75,585_01 (*) 05585_02 (*) 75,585_02 (*) 02865_01 02965_01 02865_02 02965_02	69 250 149 142 114 916 820 92 574 344 135 241 78	168 4708 2015 1978 3086 10,840 9710 4220 12,743 5294 3985 5141 4411	8:45 8:45 7:11 8:39 12:05 14:49 9:28 8:34 15:11 15:11 14:49 9:28 8:34

## 4 Experimental Design, Materials and Methods

In order to set up the experiment, the necessary steps are:

1. To create a database (MySQL/MariaDB, in our particular case) to store every event, as described above in the Data Description section.
2. To create an HTML file for each video or set of videos, with a JavaScript program to capture all events and send them to a server-side program that will then store those events in the database. This is documented in [16].
3. To create the aforementioned server-side program (in PHP, in our particular case) that receives events from the client-side program and stores them in the database. This, again, is documented in [16].

To make deployment as easy and universal as possible, we have decided to use a LAMP (Linux, Apache, MySQL, PHP) stack on the server. No additional software packages and dependencies are needed. While other stacks may be used, and may be more performant, our use scenario is easily absorbed by even very basic hardware. The versions employed in our case for Apache Server, MySQL, and PHP are httpd-2.2.15-69.el6.centos.x86_64, mysql-server-5.1.73-8.el6_8.x86_64, and php-5.3.3-50.el6_10.x86_64, respectively. We do not expect issues using more recent versions of the software, at least up to Apache 2.4.6, MySQL 8.0.42 (or MariaDB 11.7.2) and PHP 8.4.5.

To generate the data to be stored in the database, the Popcorn.js JavaScript library [17] was used, as it was one of the very few available JavaScript libraries that allowed for our intended use case at the time. Popcorn.js is an HTML5 video and media library for the web. It "allows web developers, filmmakers, artists, designers and others to easily create timeline-based web productions". Popcorn.js allows the use of videos in a wide array of formats and origins, including HTML5 video, but also videos embedded from online services such as Vimeo or YouTube. In our experiments, videos have been sourced from the department's institutional account on Vimeo, but the provided code [15] can be easily modified to use other sources. An example of a set of pages with the data collection setup with the provided code can be accessed at http://cimanet.uoc.edu/videos/bbdd/.

Popcorn.js was initially supported by the Mozilla Foundation. As it has lost that support, it might be interesting to consider other JavaScript libraries allowing the same functionalities (although it is currently actively maintained). It is convenient to use a JavaScript library to abstract us from the different player implementations in online services (such as YouTube or Vimeo). Video.js [13] and Plyr [14] are two such libraries, with good support for our needs, and well documented and maintained.

On the client side, once the page is loaded into the user's browser:

• The video is inserted into the web page using Popcorn.js.
• The initial timestamp and random key are generated.
• A pageloaded event is sent to recorder.php.
• Events are created for the video in order to intercept every instance in which the user clicks on the play and pause buttons, every time he/she scrubs through the timeline and, if an index is made available to students, every time she clicks on an index item.

Table 5 contains a fragment of the dump.csv file. For clarity, the three columns containing repeating content have been omitted: timestamp_inicial contains the “Wed, 08 May 2013 22:07:16 GMT” value, random contains the “0.21737804209767375″ and video contains the “estadistica4 value”. The sequence of events described in the table is as follows:

• The page containing the “estadistica4” video is loaded on Wed, 08 May 2013 22:07:16 GMT (this information is derived from the timestamp_inicial column mentioned above).
• At 22:07:45 the student clicks on play.
• At 22:07:53 the student clicks on pause. The playhead is at the 4.391 position. The disagreement between the playhead position and the delay of eight seconds following the previous action can be adequately explained by video buffering issues.
• At 22:08:45 the student clicks play again, with the playhead at 4.391.
• At 22:09:28 the student clicks on the timeline at the 12.688 position. There has been a 43 s delay from the previous action, so it can be assumed that the playhead was at 48.391, resulting from adding the delay to the previous playhead position.
• At 22:11:32 and 22:11:34 the student clicks on the 108.75 and 58 positions on the timeline. As the two actions are close in time, when processing the raw data, it can be assumed that both registered clicks can be considered part of a single seeking action ending at the 58 s position in the video.
• At 22:16:56 the student pauses the video at 376.334. This action takes place 5 min and 22 s (i.e., a total of 322 s) after the previous action. The playhead has moved 376.334–58 = 318.334 s, which is consistent with minimal buffering issues, and the data can be assumed as valid.
• Finally, at 22:16:57 the student clicked on play again. No further actions are recorded, so it must be assumed that either the video played until its end or that the student navigated away from the page.

**Table 5:** A fragment of the dump.csv file.

id	timestamp_accion	accion	params
319	Wed, 08 May 2013 22:07:45 GMT	play	0
320	Wed, 08 May 2013 22:07:53 GMT	pause	4.391
321	Wed, 08 May 2013 22:08:45 GMT	play	4.391
322	Wed, 08 May 2013 22:09:28 GMT	seek	12.688
323	Wed, 08 May 2013 22:11:32 GMT	seek	108.75
324	Wed, 08 May 2013 22:11:34 GMT	seek	58
325	Wed, 08 May 2013 22:16:56 GMT	pause	376.334
326	Wed, 08 May 2013 22:16:57 GMT	play	376.334

If the only intent of the web page is to record all events to a database, then the previous sequence is sufficient. On the other hand, if one wishes to trigger events (such as presenting a questionnaire to the student) as a result of a sequence of events (say, a student pauses for at least a given amount of time in a certain segment of the video, or the student skips backwards multiple times), then the sequence of events must be kept in memory in the browser and a programming logic must be created to detect the desired patterns and launch the associated actions.

In different scenarios, where the collection of personal data is allowed, both legally and ethically, the random key field could be replaced by an identifier for the user accessing the web page containing the videos. This, of course, would allow for a better understanding of behaviour across multiple sessions. This approach was not used in the data collection process for privacy and ethical reasons.

Regarding the seek action, when users scrub through the video's timeline, a rapid succession of seek events is recorded to the database, with each event having the timeline position at the moment of the event. Other collections of events that happen in short periods of time can also be considered as a single event for analysis purposes. This must be considered in post-processing, so that a single scrub having multiple recorded events is correctly interpreted. We have included the raw data, containing all consecutive events. Brinton et al. [15], for example, offer five seconds as the time period below which consecutive events should be taken as a single one.

On the server, a combination of a PHP script and a MySQL/MariaDB is used. On the database, a single table was created, described in the above section. A recorder.php file [16] is created to be called from JavaScript on the client side each time an action is performed. It connects to the database, writes a new record into it (composed of initial timestamp, random key, video, action, action timestamp and parameters) and closes the connection to the database. A more robust infrastructure may be used if there is a suspicion that the environments in which the data collection is to be used in (implementing mechanisms to ensure the events are correctly recorded to the database). If online video is to be used, it may be argued that the environment should not give reason to do so (and, in our case, the data validation processes make us believe there is no need for such mechanisms). Also, provisions should be taken to prevent third parties from writing undesired data to the database. In the scenario where the present data were collected, such a possibility was considered highly improbable, so this was not implemented. Also, instead of defining an ad hoc database schema, it may be useful to use mechanisms such as xAPI [18], "a new specification for learning technology that makes it possible to collect data about the wide range of experiences a person has". xAPI "captures data in a consistent format about a person or group’s activities from many technologies. Very different systems are able to securely communicate by capturing and sharing this stream of activities using xAPI’s simple vocabulary". The use of such solutions, though, makes deployment harder and development of ad hoc solutions slower, so their use should be carefully considered.

In order to process the data from the database, said database has been dumped as a comma-separated values (CSV) file, which can then be imported by tools such as R, Matlab, Python scripts, or even spreadsheet software such as Microsoft Excel, LibreOffice Calc or Google Sheets. The data can be exported from the database using a variety of tools, from SQL code to GUI tools such as PHPMyAdmin.

When importing these CSV files into such tools, special attention must be paid to columns containing timestamps, as these timestamps, because of the infrastructure we have used, are in JavaScript format, when most data processing tools assume timestamps are in other formats, such as Unix timestamps. In the included CSV files, timestamps have already been converted to an appropriate format. The included Python scripts contain examples of how to deal with these timestamps.

The educational videos for which playback data have been included in the present dataset are in Spanish and Catalan. In order for the dataset to be of interest to the widest possible community, they have been transcribed and made into SRT files, which have then been translated into English and made available in the same SRT format.

Naturally, extensive tests were carried out before deployment to check that data input was correctly reflected in the collected data, with no remarkable incidents detected. Beyond that, a sample of sessions are visualized regularly to check for any inconsistent data. In a number of occasions, sequences have been detected where temporal incongruence appears:

• In some cases, the point following a play action would imply that playback speed is faster than 200 %. In our data, playback at a faster than 100 % speed is extremely uncommon, and there is some evidence that very few, if any, users of online video of any kind ever go above 200 % speed.
• In some cases, a sequence of points has been detected implying a backwards move by the play head with an inconsistent displacement in the y-axis. This would imply either that a data point has been lost or that incorrect data has been input into the database.

The offending sequences have not been removed from the raw data. Code in Python has been implemented to process the data taking into account such occurrences and pruning the whole playback from the results. This code can be found in the fileprocessing2.py file in the GitHub repository [16]. In particular, we check for all timestamps to be in an ascending order (lines 73 to 79 in the code), and for discrepancies in temporal data both in pauses (lines 128 to 133) and seeking actions (lines 150 to 153).

It is important, if the data collection solution is deployed, that tests are carried out before deployment to ensure data are recorded properly and that measures are taken for random sampling of the collected data in order to visually check that it is consistent.

Basic knowledge of R, Python or other data processing tools or languages is needed in order to deal with the presented data. In, particular, in R, in order to create a dataframe representing the data for one of the videos, a command such as riak <- subset(dump, video == "riak", select = *c*("page_loaded", "rnd_key", "timestamp", "action", "param", "replayID")) may be used (which will subset the records pertaining to the riak video to a dataframe called riak containing the columns page_loaded, rnd_key, timestamp, action, param, and replayID. From this point on, basic R commands can be used to produce graphical representations for each trajectory in the dataset, to try to produce clusters for different trajectories, and so on.

## Limitations

Initial tests included a "keep alive" signal in order to better monitor where the video play head was at every five seconds. After checking that in almost all circumstances it only added non-significant records to the dataset, it was removed. If the data collection was to be replicated in environments with unreliable or untrusted internet connections, reinstating that "keep alive" data is recommended. This “keep alive” signal would also be useful to determine whether “navigated away” events are not being properly detected, or there are other issues preventing the accurate detection of video playblack dropping out for any unknown and currently undetected issues. Also, schemes for more reliable data collection (with strategies to keep data in the client, ping the server and ensure the data has been stored correctly) may be implemented. In general, though, it is to be expected that if online video is used as a learning resource, the available infrastructure should be robust enough so that this kind of measures is not required.

Also, no provision has been made to check whether users are using different video playback speeds. There is almost no evidence in the dataset that students change playback speed (this can be inferred from consecutive data points in the dataset), but it would be interesting to check for those changes if data were to be collected in other environments. Different strategies can be used to do so, including both detecting the change of playback speed event and the already mentioned "keep alive" signal (measuring the differences in playhead position at regular intervals to independently calculate playback speed).

Also, attention must be paid to the fact that timestamps are generated locally in the browsers of users accessing the webpages. While this is infrequent, this may result in erroneous timestamps. For the purpose of our work timestamps are only used in a relative manner (that is, only the difference between the initial timestamp and subsequent ones in a session are of interest), so no further measures have been taken in order to ensure accurate timestamps. If this was needed, it may be of interest to generate a second timestamp on the server side. Also, because of the sequential nature of the stored data, timestamps inaccurate to a big degree (days) should be easy to detect in the data. No such timestamps have been detected in the present dataset.

## Ethics Statement

The data collection that has given as a result the present dataset has been validated by the ethical committee at the Open University of Catalonia, complies with regulations such as the European General Data Protection Regulation and is completely anonymous. The collected data are covered by the current privacy and data collection agreement students agree to when accessing the Open University of Catalonia’s virtual learning environment, and thus no additional agreements are required.

## Credit Author Statement

César Córcoles and M. Antonia Huertas conceived and conducted the experiments that produced the collected data. César Córcoles, Germán Cobo Rodríguez, Ana-Elena Guerrero-Roldán and M. Antonia Huertas analysed the results. César Córcoles wrote the manuscript and all authors reviewed and edited it.

## Data Availability

ZenodoA dataset on the use of online video by students at the in-video level (Original data).
